# Flexure Performance of Textile-Reinforced Cementitious Composites with Novel Inclined Reinforcements

**DOI:** 10.3390/ma17194743

**Published:** 2024-09-27

**Authors:** Esat Selim Kocaman, Thomas Henzel, Olcay Gurabi Aydogan, Can Gurer Yucel

**Affiliations:** Department of Civil Engineering, Bogazici University, Istanbul 34342, Türkiye

**Keywords:** cementitious composites, smart material design, material modeling, strengthening, textile-reinforced mortar

## Abstract

Textile-reinforced cementitious composites have great potential to offer novel design opportunities for thin-section structures thanks to their superior material capabilities. In this work, new cementitious composites with novel reinforcement configurations are developed, which have superior mechanical properties. The cementitious composites contain inclined through-the-thickness reinforcements, and their enhanced performance on thin-section material hardening under flexural loading is demonstrated. Furthermore, a new practical FE modeling approach is proposed that involves the combined use of multiple cohesive regions and 1D reinforcement elements that pass through these regions with a bilinear material law. This approach provides a new computationally efficient modelling framework whereby reinforcement pull-out during hardening is readily captured without resorting to computationally demanding interface laws between the reinforcement and the cementititous matrix. The model can model enhanced hardening of new configurations and provides comparable results with the experimental findings. The model can be used in the modelling and design of novel cementitious composites with engineered reinforcement configurations. Overall, this study aims to open up new avenues for the smart material design of cementitious composites with novel structural reinforcements.

## 1. Introduction

### 1.1. Background and Motivation

Textile-reinforced cementitious composites in the form of Textile-Reinforced Concrete and Mortar (TRC and TRM) are increasingly used in various applications thanks to their superior mechanical performance [1,2,3]. These are novel composite materials consisting of textile reinforcements (typically in the form of polymer-coated carbon fiber or glass fibers) embedded inside concrete or cementitious materials. Textile reinforcements can significantly increase ultimate strength and ductility of concrete [1,2,3]. They are used in realizing components with improved load-carrying capacity [4,5,6,7,8,9]. They have been used in a variety of applications to produce thin-section structural parts with aesthetic appeal and enhanced durability [2,3,4,5,6,7]. These applications include bridge decks, architectural elements, and precast concrete components [1,2,3,4]. Another critical application of these cementitious composites is in the strengthening of aging structures, which has also gained great importance and a found wide range of applications [5,6,7,8,9,10,11,12,13,14]. Through engineered material design, TRC can provide non-structural benefits for structures with improved energy efficiency and fire resistance for architectural elements [7]. Various experimental works were conducted to better understand the hardening of reinforcements under different loading conditions, and researchers have proposed new modeling approaches to capture their material behavior, particularly for their hardening [15,16,17,18,19,20,21,22,23,24,25,26]. Environmental effects, particularly temperature, can have a strong influence on TRC and TRM properties, which have also received significant research attention [27]. In different works [17,18], the reinforcements were modeled with 1D elements and embedded inside a cementitious matrix. As this matrix experienced failure based on a damage criterion, this failure was modeled in a smeared manner using damage models by reducing the stiffness of the failed regions. Furthermore, the interaction between the reinforcement and the cementitious matrix is of great importance, as it has significant influence on the overall mechanical properties [18,21,23,24]. Various tests and models have been proposed to better understand this interaction and capture the material hardening behavior effectively. It is vital to explore new practical modeling approaches that can readily capture the physical characteristics of textile-reinforced cementitious composites and to provide computationally efficient yet detailed hardening models for TRC structures with different reinforcement configurations.

The application of textile-reinforced cementitious composites in construction is not well-established, which demands further research to fully understand their performance, especially for thin-section structures. Crucially, the development of various reinforcement configurations on cementitious composites is important in realizing smartly configured TRCs with more effective reinforcements. This work explores new ways of reinforcing cementitious matrices and new reinforcement mechanisms. It aims to understand how they can be engineered to improve the overall properties of textile-reinforced cementitious composites. It proposes novel reinforcement configurations for the design of textile-reinforced composites with improved mechanical performance. A preliminary work towards this vision was conducted by the authors in [25]. In this work, new reinforcement configurations, together with a more comprehensive experimental and numerical analysis, were provided. Furthermore, a novel practical modeling approach was also developed to improve the understanding of these new materials systems with different reinforcement configurations. Our method proposes the concurrent use of cohesive elements and 1D reinforcement elements that pass through them with a bilinear material law. The method captures the physical characteristics of these new material systems and provides numerical explanations for the behaviors of different reinforcement mechanisms. The model involves direct explicit modeling of crack discontinuities, contrary to the smeared approaches found in the literature, and provides a practical approach to model material hardening. The bilinear materials law enables modeling of the hardening without the need for complicated slip laws between the textile reinforcements and the cementitious matrix, as required by the models in the literature [17,18]. This can provide a computationally efficient framework for a more detailed analysis of large-scale thin-section structures when considering their failure, whereby crack discontinuities are explicitly modeled. Overall, the main goal of this work is to develop new textile-reinforced cementitious composites with through-the-thickness reinforcements for their enhanced mechanical performance. It also aims to provide a practical modeling framework for the analysis and design of smartly configured cementititous composites.

### 1.2. Outline

In this paper, we first present the methodology for the work in Section 2, where we explain the developed reinforcement configurations, their preparation, and testing (Section 2.1), as well as numerical modeling (Section 2.2). The proposed modeling approach to capture the flexural behavior for the proposed configurations is explained in detail in Section 2.2. This section is followed by Section 3, where we present the experimental and numerical results for the hardening behavior of the configurations. Vital insight and discussions regarding the nature of inclined reinforcements and their modeling with new approaches are presented in this section. The paper is finalized with the overall conclusions in Section 4.

## 2. Methodology

### 2.1. Preparation and Testing

**Figure 1:**
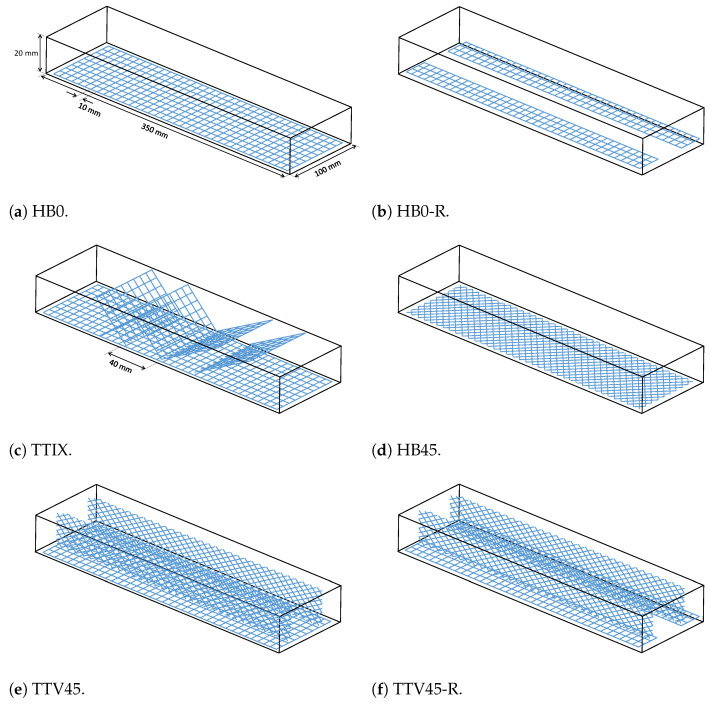
Schematics for the tested reinforcement configurations.

The casting and testing for textile-reinforced cement specimens with different configurations are explained in this section. Materials used to produce cement-based composite were Portland cement, tap water, sand, and a polycarboxylate superplasticizer (SP). Portland cement used in this study was CEM I 42.5 R with TS EN 197-1 [28] standard. Natural sand was used with a maximum particle size of 2 mm. Textile reinforcement (Figure 1a) consisted of grid-shaped polymer-coated glass fiber bundles (fiber-reinforced polymer composite) in two perpendicular weft and warp directions with same areal weight (plain weave bidirectonal grid with approximately 320 $g/m^{2}$ areal density). The spacing, thickness, and width of the grids were approximately 10 mm, 0.3 mm, and 3 mm, respectively. With these textile grids, specimens with various reinforcement configurations were produced. The schematics for various configurations of reinforcements are provided in Figure 1, which shows the spatial positioning of textile grids inside the specimens.

To ensure effective compaction of textile reinforcement inside specimens during casting, the cementitious mixture must ideally have sufficient viscosity such that the mixture can pass through the textile grids and adequately cover them. Towards this end, we selected a mixture having a water-to-cement ratio of 0.45 and a sand-to-cement ratio of 1.5 as the baseline for all specimens. We conducted Flow table tests, which showed an average diameter of 21.57 cm (using the standard ASTM C230/C230M-14 [29]). This mixture exhibited sufficient workability for the practical placement and compaction of the textile grids inside the cementitious matrix during casting.

After this stage, we prepared the textile reinforcements for the different configurations of specimens. Figure 1a shows the dimensions of the baseline textile grid reinforcement. For this work, 5 different reinforcement configurations were tested in addition to the baseline horizontal reinforcement configuration HB0. These configurations are illustrated in Figure 1.

Baseline specimen HB0 had horizontal textile reinforcements 2 mm above the bottom specimen surface, as shown in Figure 1a. Specimen HB0-R had reduced horizontal reinforcement configuration, where one third of the horizontal reinforcement at the middle section was removed along the specimen length (see Figure 1b). It contains reinforcements close to the specimen edges along the length direction. The effect of this material removal on the overall behavior was of particular interest to shed more light on the reinforcement mechanisms and associated hardening. Specimen HB45 had horizontal textile reinforcements 2 mm above the bottom specimen surface, with a grid orientation of 45° (see Figure 1d).

Among the new configurations, the TTIX has additional inclined reinforcements placed along the specimen thickness oriented 45° with respect to the thickness direction (see Figure 1c). This is to reinforce the cementitious matrix to resist and bridge shear cracks upon flexural loading to increase the hardening of the textile-reinforced composite. This configuration is referred to as TTIX (through-the-thickness inclined cross). A preliminary work on new reinforcements was presented by the authors in [25]. Furthermore, another new configuration (see Figure 1e) was also developed where, in addition to the grid reinforcements with 0° orientation, two textile reinforcements with a grid orientation of 45° (similar to HB45) were placed close to the specimen edges vertically along the thickness direction of the specimen. This is to initiate crack bridging throughout the flexure direction of the specimen. This configuration is named TTV45 (through-the-thickness vertical reinforcement with 45° angled grid reinforcements). The configuration TTV45-R is the reduced version of TTV45 where, again, one third of the horizontal reinforcement at the middle section was removed along the specimen length (see Figure 1f). For configurations TTIX, TTV45, and TTV45-R, the through-the-thickness reinforcements have textile grids oriented at 45° with respect to the thickness direction. Thus, these reinforcements are all referred to as inclined reinforcements throughout this study.

For each configuration, we tested 3 specimens except for the baseline HB0 and TTIX configurations, for which 4 specimens were analyzed. For specimen TTV45, 2 specimens were considered, as one of the specimens experienced a manufacturing problem involving configuration placement. Before casting, the textile reinforcements was cut based on the spatial configurations of the specimens. After this preparation, the portion of the mixture corresponding to the 2 mm specimen thickness was poured into the mold. Then, the horizontal reinforcements were placed. After this step, the remaining volume corresponding to the 18 mm thickness was filled with the mixture in two steps. At each step when a portion of the mixture was poured into the mold, we applied vibration for effective cement compaction. For the configurations TTIX, TTV45, TTV45-R, the inclined reinforcements were placed with the help of support sticks carefully positioned inside the mold. For real life applications, through-the-thickness reinforcements can similarly be held from above the mold with positioners; then, the casting can be performed gradually, where vibration can be applied at each stage for effective compaction. Structural reinforcements with various configurations can then be successfully produced.

Furthermore, to verify that the mixture provided a sufficient compressive strength, compression tests were carried out with 4 × 4 × 4 cm cubic specimens using an electro-mechanical compression testing machine. We cured the compression and bending specimens at 21.2 °C and 95% relative humidity for 28 days.

In this work, new configurations were studied to explore new reinforcement mechanisms for improved mechanical performance of thin-section TRCs. To evaluate the hardening of these new configurations, three-point bending tests were conducted on thin-section specimens. The specimen dimensions were 35 cm in length, 10 cm in width, and 2 cm in thickness. A large support span with a length of 27 cm was selected to simulate severe bending conditions for testing. Customized dimensions were chosen to evaluate the behavior of different reinforcement configurations in a representatively large region that was exposed to severe bending.

**Figure 2:**
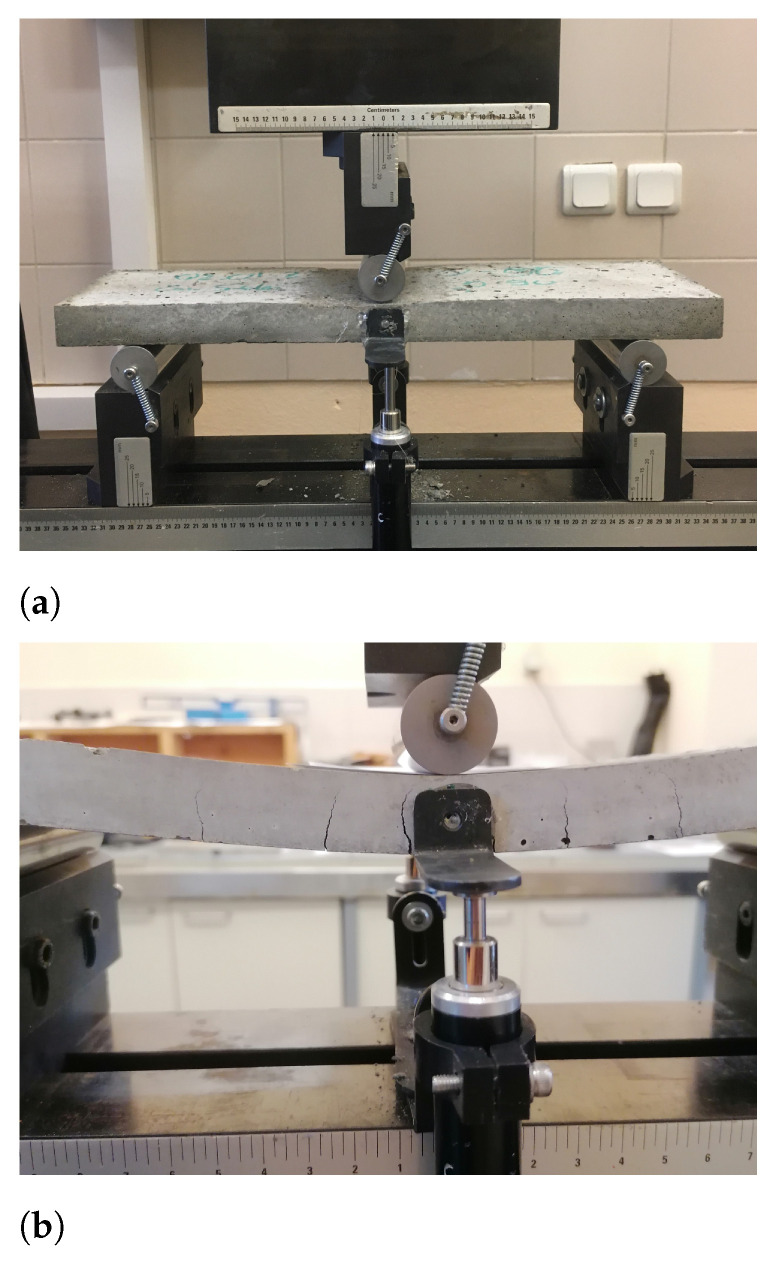
Three-point bending test setup with the supports and LVDT installation for displacement measurement. (**a**) Testing setup; (**b**) Zoomed specimen and formation of various cracks.

**Figure 3:**
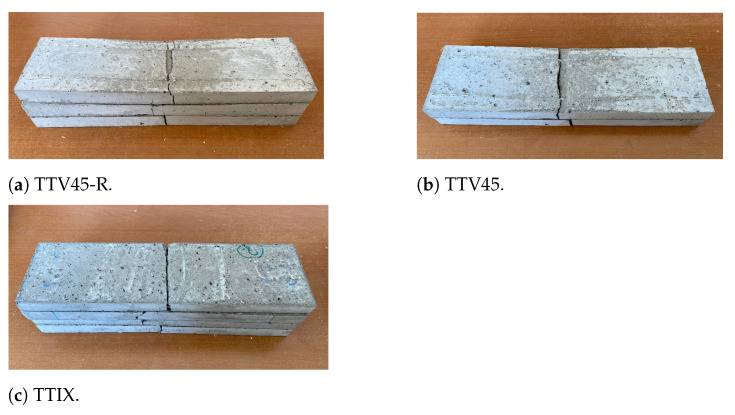
Specimens with inclined reinforcements after testing.

The flexural tests were conducted using an MTS servo-hydraulic testing machine in displacement-controlled mode with a rate of 2 mm/min. Figure 2 shows the three-point bending testing setup. To measure the specimens’ midspan deflection, two linear variable displacement transducers (LVDTs) were mounted on both sides of the specimens. The tested specimens are provided in Figure 3.

Upon manufacturing, specimens had slight variations in their thickness, which were calibrated and normalized by finding the effective stress and strain of the specimen at the lowest point of the middle section. Using the force and displacement of the specimens, the stress and strain curves of the specimens were determined using the following equations [30]:

(1) $$\sigma_{f}=\frac{3FL}{2bd^{2}},$$

(2) $$\epsilon_{f}=\frac{6Dd}{L^{2}},$$

where *d* is the beam thickness, *b* is the beam width, *L* is the span length, *F* is the flexural load, *D* is the beam midpoint maximum deflection, $\sigma_{f}$ and $\epsilon_{f}$ are the stress and strain at the bottom surface center, respectively.

### 2.2. Model Development

To numerically capture the experimental findings on the reinforcement behavior, 2D FE models were developed. The objective of these FE models was to provide a new modeling approach for smartly configured textile-reinforced cementitious composites, as well as to provide important numerical insights on the observed superior hardening for the inclined reinforcement configurations.

**Figure 4:**
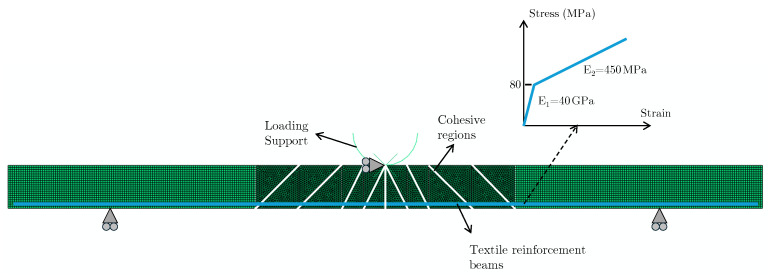
Schematic of the FE model (The cohesive regions and reinforcements are shown with white and blue color, respectively); bilinear materials law assigned to the reinforcements is also shown.

Abaqus 2023 Finite Element software was used for the modeling of the three-point bending tests. In the models, the thin-section specimens with dimensions 350 mm × 100 mm × 20 mm were modeled using 2-dimensional plane strain continuum elements along the thickness direction, as shown in Figure 4. The textile reinforcements were modeled with 1D beam elements and embedded at the appropriate locations along the specimen thickness and length based on the simulated reinforcement configuration. The mesh involved both triangular and quadrilateral elements for the continuum parts, and a refined mesh with an average element size of 1 mm was selected both for the cementitious matrix and reinforcement sections to ensure the sufficiency of the mesh.

**Figure 5:**
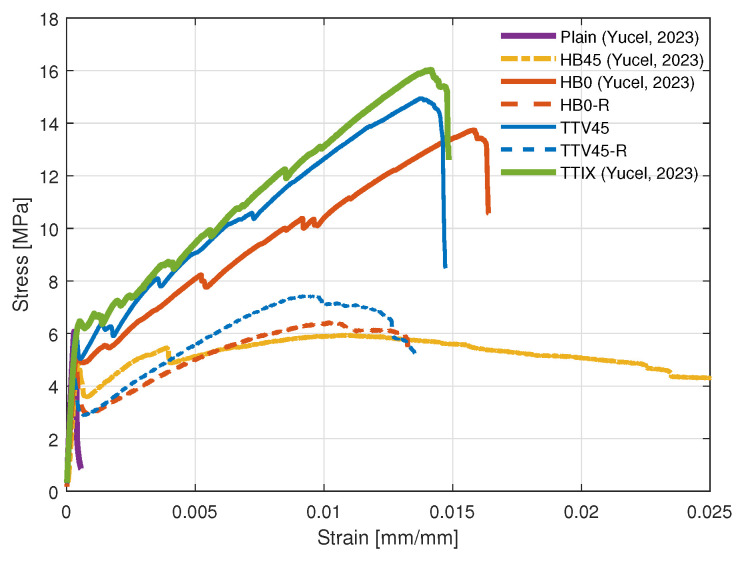
Stress–strain curves of cementitious composites with different reinforcement configurations under three-point bending, referenced data are from [25].

Considering the bending of thin-section cementitious mortars, they fail in a brittle manner at low deformations without experiencing significant stresses (see Figure 5 for plain specimens corresponding to the mortar with no reinforcements). This material condition until failure can be idealized as an elastic behavior. On the other hand, in the case when there are reinforcements inside a cementitious matrix, the load is transferred to reinforcements after the sudden brittle failure of the matrix at low deformations of flexural loading. The reinforcements then continue to carry the load, whereby material can experience significant deformation. The high deformation is achieved by cracking of the matrix and the pull-out of the reinforcements from these crack surfaces as they exhibit crack bridging. This introduces a pseudo-plastic behavior to the TRCs, whereby hardening occurs, and load capacities of the TRCs are significantly increased (see Figure 2 configuration).

This hardening behavior of the cementitious composites is dictated by both the elasticity of the reinforcement and its pull-out from the cementitious matrix. This pull-out or slip region corresponding to the reinforcement and the matrix interaction can have a strong influence on the hardening behavior of cementitious composites [11,18,21]. Cumulative effects of both the textile reinforcement and its interaction with the matrix are primarily responsible for the overall hardening behavior.

Based on these considerations, an elastic material behavior was assigned for the cementitious matrix of the specimens in the FE model. Isotropic elastic properties were selected for the material behavior of the solid matrix section with a Young’s modulus of 20 GPa and Poisson’s ratio of 0.2. The loading section was modeled with discrete rigid elements, which are applied displacements for quasi-static analysis of the three-point bending test. Hard contact conditions were assigned between the specimen and the loading section. The span supports at the bottom were simulated with fixed boundary conditions on the vertical displacements. Furthermore, the loading support and the top nodes at the middle of the specimen touching the support were also clamped in the horizontal direction.

To simulate the reinforcement effect on the material hardening, a simple methodology was proposed. First, a cohesive region was introduced to the middle of the specimen spanning the whole thickness beneath the loading point. The cohesive elements along this middle crack have low tensile strength and fracture energy (0.5 MPa and 0.075 $\mathrm{kJ}/m^{2}$), so they fail at low deformations. A reasonable set of values was used to simulate the brittle nature of cementitious composite failure based on the baseline HB0 stress–strain curve. The assigned cohesive properties are provided in Table 1. In addition, three cohesive surfaces were added to each side of the specimens to represent the inclined shear cracks that occur upon bending, as shown in Figure 4. These cracks can occur at various locations and angles along the specimen upon flexural loading. These cracks are collectively represented with the addition of these cohesive elements, as shown in Figure 4. Crucially, the 1D beam elements of the reinforcements pass through these cohesive regions. After their failure, these embedded 1D beam elements that pass through the crack surfaces continue to carry the flexural loads, whereby hardening stage are simulated. Essentially, after cohesive failure, the disconnected pieces of the specimen are held together by the reinforcements, which carry the load and provide the structural integrity.

To capture the cumulative effect of the reinforcement and pull-out during hardening, the beam elements of the reinforcements were assigned a bilinear material law (see Figure 4). The bilinear behavior was proposed based on the considerations of the aforementioned material behavior and the experimental results. The initial linear region of the bilinear law corresponds to the unfailed stage of the specimen, where perfect bonding of the reinforcements with the cementitious matrix can be assumed. The reinforcement stiffness contributes fully to the specimen, as they deform together upon flexural loading. The second line corresponds to the stage where the cohesive regions fail, and the matrix no longer carries the load. Instead, it is then primarily carried by the reinforcements, as they exhibit crack bridging and fiber pull-out. This stage, which stems from the interaction of the reinforcements with the matrix, is then simulated with a second elastic regime in the stress–strain curve of the reinforcements after the first linear stage. This cumulative interaction at the second stage results in a different effective stiffness for the reinforcements. The introduction of cohesive regions with low fracture properties and a second elastic regime for the reinforcements are critical for computationally efficient hardening modeling without resorting to computationally demanding cohesive interface laws between the reinforcements and the cementitious matrix.

The reinforcement grids containing glass fibers embedded inside a polymer coating were modeled using 1D beam elements with a Young’s modulus of 40 GPa and Poisson’s ratio of 0.3. These properties correspond to the initial linear stage of the bilinear material law. The second linear line starts at 80 MPa and has a modulus of 450 MPa (see Figure 4). This yield strength and hardening modulus (slope of the second linear line) of the bilinear material law were determined based on fitting to the experimental results of the baseline configuration HB0. This bilinear law was also assigned to the inclined reinforcements of the configuration TTIX to simulate their hardening behavior and capture the experimental results for verification of the model. The strain at maximum stress of HBO (0.0158 mm/mm) was used as the failure point of reinforcements and correspondingly for the specimens for the models of other configurations (TTIX). When assigning the beam elements, their profiles were determined based on the number of horizontal grids passing through the specimen along the bending direction and their cumulative cross-sections. Considering the topology of the configurations, this 2D model was only applied to configurations HB0 and TTIX, as their 2D idealization was plausible. However, this modeling approach can be extended to 3D, and more complex 3D configurations such as TTV45 can be readily modeled. This 3D extension of the model has been left to the scope of future work on these material systems.

After the development of the 2D FE models, we conducted a quasi-static numerical simulation and determined the stress–strain curves of the specimens with different configurations, which were determined from force–displacement data using Equations (1) and (2) for comparison with the experimental results.

## 3. Results and Discussion

The average compressive strength of the specimens was determined as 37 MPa. A reasonable value was attained, and this mortar was selected as the matrix for the subsequent composite specimens. For all the configurations, the average stress–strain curves of the flexure testing are plotted in Figure 5. For configurations exhibiting hardening (HB0, TTIX, and TTV45), the critical strength and modulus results are provided in Table 2. The hardening modulus values were calculated based on linear fitting to the start and end of the second stage in the stress–strain curve.

The configuration HB0 (see Figure 5) exhibited clear hardening in line with the findings in the literature [9]. The stress–strain curve before the ultimate strength consisted of two stages. The first stage entailed the initial elastic region until the major matrix failure at low flexural deformations, and the second stage covered the subsequent region until the ultimate strength. Considering the behavior of these two regions, they can be idealized as linear curves, with each having a different associated modulus. As explained in Section 2.2, these two linear regions stem from the matrix failure and subsequent reinforcement pull-out. The equivalent stiffness and starting yield stress values of this second stage can be determined based on fitting to the experimental results of the baseline configuration HB0. After reaching the ultimate strength, the stress dropped dramatically in several steps corresponding to severe reinforcement pull-outs and breakage, whereby the specimen lost its structural integrity. The configuration HB45 (Figure 1d) with off-axis grid reinforcements did not exhibit hardening behavior; however, they had increased ultimate failure strain compared to the configuration HB0 with 0° grid orientation. This was due to significant pull-out of the fabrics that continued until large deformation of the specimen occurred without increasing the load carrying capacity. This same orientation of reinforcements was then used vertically in the development of the configuration TTV45 (Figure 1e).

The TTIX configuration with inclined reinforcements through the thickness showed considerably improved hardening compared to the baseline configuration HB0 (see Figure 5). Additional inclined reinforcements increased the ultimate strength and hardening modulus by approximately 17% and 31%, respectively. The through-the-thickness inclined reinforcements provide considerable enhancement in the cementitios matrix shear resistance. When shear cracks are formed, these inclined reinforcements initiate crack bridging significantly contributing to the material hardening. Cumulatively, these effects boost the hardening and strengthening of the cementitious composite in a substantial manner.

In configuration TTV45, in addition to the baseline horizontal reinforcements, there were vertical shear reinforcements along the specimen length oriented at 45° with respect to the thickness direction (see Figure 1e). Compared to the TTIX configuration, these reinforcements did not span the whole width of the specimen and rather spanned the whole length. With the addition of these reinforcements, the hardening behavior notably changed. The hardening strength and the modulus were increased in average by 10% and 27%, respectively. With this configuration, considering that the inclined reinforcements span the whole length, crack bridging can be achieved throughout the specimen flexural direction, as the cracks can occur at any location along the specimen. Even though these reinforcements are not fully aligned in the flexure direction, and they reinforce the specimen only at two points along the width, they can still stiffen the cement matrix and contribute to the hardening in a noteworthy manner. As in the TTIX configuration, this configuration also demonstrated the considerable effect of the inclined reinforcements in the hardening of thin-section cementitious composites. The TTIX configuration showed superior mechanical properties compared to the TTV45, as more crack bridging was initiated along the major middle cracks; however, the TTV45 configuration has the capability of bridging all the inclined cracks that can occur at any location along the specimen upon flexural loading.

The HB0-R configuration with removed reinforcement middle section exhibited considerably different hardening behavior compared to the baseline HB0 configuration. Removal of one third of the reinforcements from the middle section (see Figure 1b) did not result in a reduction in the hardening comparable to this removed material percentage (33%). Rather, a dramatic reduction in hardening was detected (Figure 1b). This can be attributed to the stress concentration on the remaining reinforcement materials close to the specimen edges, causing earlier failure of these textiles and the specimen. The stress transfer to the reinforcements was affected, and the hardening modulus that originates from textile reinforcement and its connection to the cement matrix substantially decreased. Thus, the observed hardening changed significantly. Similarly, the configuration TTV45-R with a removed reinforcement middle section exhibited substantially different hardening behavior compared to the TTV45 configuration. Again, removal of the middle part had a considerable effect on the overall hardening. Furthermore, for the reduced configurations, the thinner reinforcements along the edges of the specimens (in the flexure direction) can have a weaker anchorage to the cementitious matrix compared to the other configurations, where reinforcement spans the whole specimen. Anchorage to the matrix is reduced with the reinforcement removal from the middle section. This can cause increased reinforcement sliding starting from the early deformations, which leads to more non-linearity in the stress–strain curve.

The hardening for the reduced configurations became limited compared to the plain specimens (5% and 15% increase for HB0-R and TTV45-R, respectively). Our study explored the possibility of less reinforcement material use for a similar hardening performance and for these configurations; the results suggest that hardening can be rather sensitive to the number of reinforcement grids and the existence of small unreinforced regions. For a simple flexure of thin-section cementitious composites, the existence of narrow unreinforced regions can dramatically reduce the hardening capacity and damage tolerance. On the other hand, compared to the other reduced HB0-R configuration, the TTV45-R exhibited a clear increase in its hardening modulus and strength (see Figure 5). This again demonstrated that the hardening capacity increased with the inclined reinforcements.

**Figure 6:**
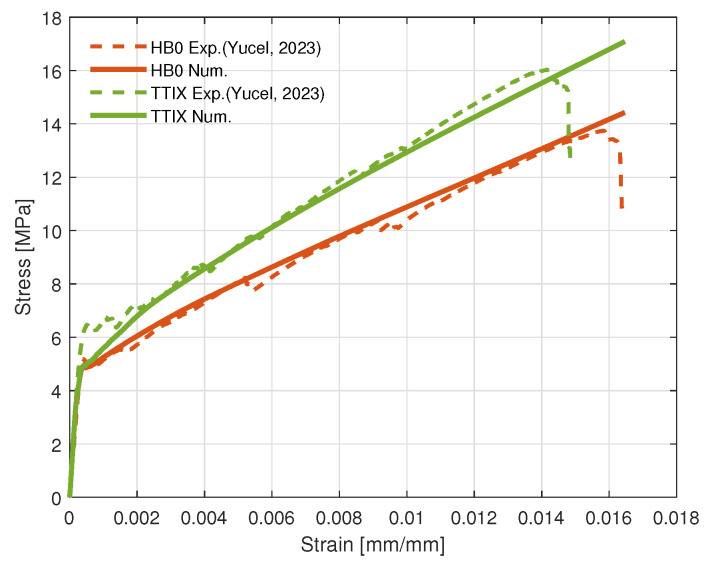
Comparison of experimental and numerical stress–strain curves for different configurations, referenced data are from [25].

**Figure 7:**
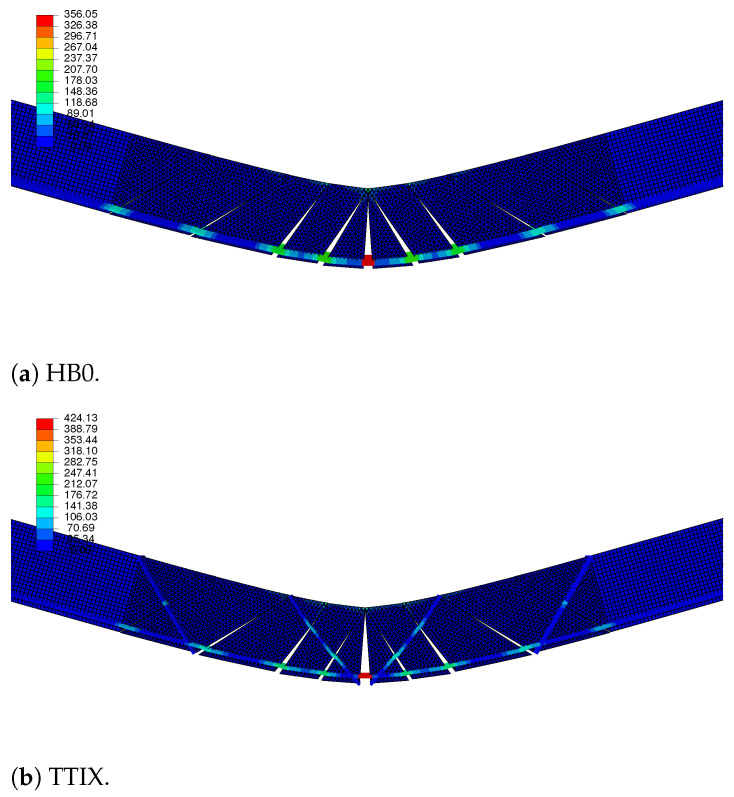
Deflection of different configurations with failed interface cracks and pulled-out textile reinforcements (Color bar shows the von Mises stress distribution along the reinforcements in MPa).

The stress–strain curves of the FEM models are provided in Figure 6 for the HB0 configuration and TTIX configuration. In addition, the deformation plots of these configurations and stress distributions, particularly along the reinforcements, are given in Figure 7. Overall, the stress–strain curves of the numerical models for the HB0 and TTIX have good agreement with the experimental findings. Using the experimental results of the HB0, parameters for the bilinear material law corresponding to the cumulative effect of the reinforcement and pull-out region were extracted and supplied to the FE model. With this approach, the HB0 model was able to capture the fundamental characteristics of the material behavior and the experimental results (see Figure 5). This bilinear material law was then supplied to the inclined reinforcements of the other configurations with their corresponding cumulative beam cross sections.

Overall, the stress–strain curves of the numerical model for the inclined TTIX configuration are in good agreement with the experimental findings. The initial transition region occurring at very low deformations can be rather challenging to capture when considering the brittle nature of the TRM, and this was not the focus of this study nor of the model. For the TTIX configuration, the results capture the steeper trend of the hardening and higher ultimate strength of this configuration. These results indicate the crucial importance of inclined crack bridging at multiple locations of the crack surfaces, as well as bridging a greater number of cracks throughout the material in increasing the hardening capacity of cementitious composites. Through the proposed model, this observation was also verified numerically. More accurate 3D models would enable the simulation of more complex 3D configurations such as TTV45, which has been left to the scope of future work on these material systems.

The deflection and von Mises stress distribution plots of the configurations show the reinforcements pull-outs from the crack surfaces (Figure 7). The resultant stresses along the reinforcements due to the load transfer after cohesive region failure can also be observed. The disconnected pieces of the specimen after matrix failure were held together by the reinforcements, which carried the flexural loads. The cementitious matrix part experienced a notable stress; insteadm the load was carried by the reinforcements (which experienced the greatest stress along their length), as the cohesive regions failed at low deformations of flexural loading. The inclined reinforcements of the TTIX configuration also experienced considerable stresses, increasing the shear resistance and hardening capacity of the cementitious composite.

Exploring the combined use of cohesive regions and 1D reinforcements with a bilinear material law as a new modeling approach for cementitious composites revealed key insights with respect to the hardening and enhanced capabilities of the new reinforcement configurations. This practical approach can be used in the construction of larger models that contain explicit discrete cracks for the more accurate simulation of larger thin-section structures. The methodology can enable computationally efficient modeling of large cementitious composite structures in comparison to their more detailed high-fidelity modeling counterparts.

## 4. Conclusions

In this work, new cementititous composite materials were developed with novel reinforcement configurations. The developed configurations, namely, TTIX and TTV45, with through-the-thickness inclined reinforcements improved the hardening capacity of the textile-reinforced composites in a considerable manner. This suggests that inclined reinforcements increase the resistance to shear failure by initiating crack bridging and reinforcement pull-out, contributing to thin-section cementititous composite hardening. The significant effect of through-the-thickness reinforcements on thin-section cementitious composites has been explored and demonstrated with this work.

Furthermore, a new practical modeling approach has been proposed for the smart material design of cementitious composites. The model combines multiple cohesive regions and 1D reinforcements with a bilinear material law that passes through them to capture the cementitious composite hardening. It requires an experimental stress–strain curve of an HB0 configuration for parameter determination of the reinforcement elastic contribution, which can vary for different reinforcement and matrix material systems. It can then capture the increased hardening of new cementitious composites with through-the-thickness reinforcements and give comparable results with experimental findings. The model can provide an efficient modeling framework for engineering analysis and for the design of smartly configured cementitious composites with improved mechanical performance.

The developed new configurations can be utilized in critical regions of thin-section structures to enhance their load carrying and energy dissipation capacity. Overall, this work aims to open new avenues for the realization of cementitious composites with novel structural reinforcement configurations.

## Figures and Tables

**Table 1:** Critical cohesive and reinforcement material properties.

Property	Values
Reinforcement Yield strength	80 MPa
Reinforcement Hardening Modulus	450 MPa
Cohesive Fracture Strength	0.5 MPa
Cohesive Fracture Energy	0.075 $\mathrm{kJ}/m^{2}$

**Table 2:** Average hardening strength and modulus for the tested configurations based on fitting to the experimental data.

Configuration	Ultimate Stress (MPa)	Hardening Modulus (MPa)	Reinforcement Weight (g)	R-Squared Values
Plain	6.07	-	-	-
HB0	13.73	577	11.25	0.996
TTV45	14.94	733	15.95	0.995
TTIX	16.02	754	14.97	0.999
HB0-R	6.40	383	7.7	0.971
TTV45-R	7.43	553	12.4	0.999

## Data Availability

The datasets presented in this article are not readily available, because the data are part of an ongoing work.
